# The hormonal composition of follicular fluid and its implications for ovarian cancer pathogenesis

**DOI:** 10.1186/1477-7827-12-60

**Published:** 2014-07-06

**Authors:** Megan M Emori, Ronny Drapkin

**Affiliations:** 1 Department of Medical Oncology, Dana Farber Cancer Institute, Harvard Medical School, 450 Brookline Avenue, Boston, MA 02115, USA

**Keywords:** Follicular fluid, Ovarian cancer, Hormones

## Abstract

Ovulation has long been associated with an increased risk in ovarian cancer, yet the underlying molecular mechanisms remain obscure. Two aspects of ovulation have been linked to ovarian cancer pathogenesis. The first is the impact of repetitive tissue injury and repair that occurs with each ovulatory event. The second is the release of follicular fluid that accompanies the follicular rupture and its effect on the ovarian and fallopian tube epithelial cells. Hormones are an important component of follicular fluid, which transiently bathes the ovarian surface and fallopian tube epithelium during ovulation. Much work has been done exploring the role of hormones in fertility, but some, such as estrogen, have also been implicated in the pathogenesis of ovarian and other cancers. Understanding the role of hormones within follicular fluid, as well as how they are altered in disorders which increase ovarian cancer risk, will enhance our ability to assess risk and develop preventative strategies. This review provides an in depth discussion of the logistics of using and studying follicular fluid in ovarian cancer research, and discusses the fluctuations in follicular fluid hormone levels during normal physiological processes versus conditions that increase ovarian cancer risk.

## Background

Despite our growing understanding of the cancer genome and the evolution of targeted therapies, ovarian cancer remains the most lethal gynecological malignancy in the Western world [1]. The clinical outcomes for this disease have not changed significantly over the past four decades, in large part due the lack of early detection tools and the almost inevitable emergence of chemo-resistant disease [1]. Adding to the complexity of this cancer is its heterogenous nature. Although ovarian tumors can arise from three different cell types: epithelial cells, germ cells, and sex cord stromal cells, the vast majority of ovarian cancers are epithelial in nature. Even within epithelial ovarian cancers there exist various histologic subtypes and molecular subgroups. This has prompted the classification of epithelial ovarian cancers into two groups. Type I tumors, clearly linked to ovarian precursor lesions, encompass all histologic subtypes including low grade serous, endometriod, mucinous, and clear cell carcinomas. They are defined by their slow growth and multiple genetic mutations. In contrast, Type II tumors are highly aggressive, confer a much poorer prognosis, and many have been linked to precursors arising from the fallopian tube epithelium. High Grade Serous Ovarian Cancer (HGSOC) is the most common of the Type II tumors. Type I and Type II tumors are also genomically distinct. Type I tumors are frequently associated with specific mutations in oncogenes such as *k-RAS* and *ARID1A*[2]. In Type II tumors, *TP53* is mutated in the vast majority of tumors (96-100%) and appears to be the earliest genetic event in HGSOC. *BRCA1* and *BRCA2* mutation carriers are particularly susceptible to Type II tumors [2,3].

While much work has been done to characterize the pathology and genetics of ovarian cancer, we still lack a basic understanding of the early events and causes of this disease. Our current understanding of the risks factors of HGSOC derives primarily from epidemiological data. Lifetime ovulation is positively correlated with HGSOC, and factors such as parity and birth control, which decrease lifetime ovulation, have a protective effect against HGSOC [4,5]. Establishing a molecular mechanism linking ovulation and HGSOC pathogenesis is critical to developing screening techniques and treatments for this disease.

## Models of ovarian cancer pathogenesis

The origins of ovarian cancer are complex and still under debate. New theories suggest that different ovarian tumor subtypes have different origins, with the ovarian surface epithelium implicated in Type 1 tumors and fallopian tube secretory epithelial cells implicated in high grade serous ovarian cancer [2,3,6]. The adherent mesothelium of the ovary attracts both shed fallopian tube cells as well as endometriosis-derived Müllerian tissue, further blurring the lines of the ovary vs. Müllerian tissue debate [7].

**Figure 1:**
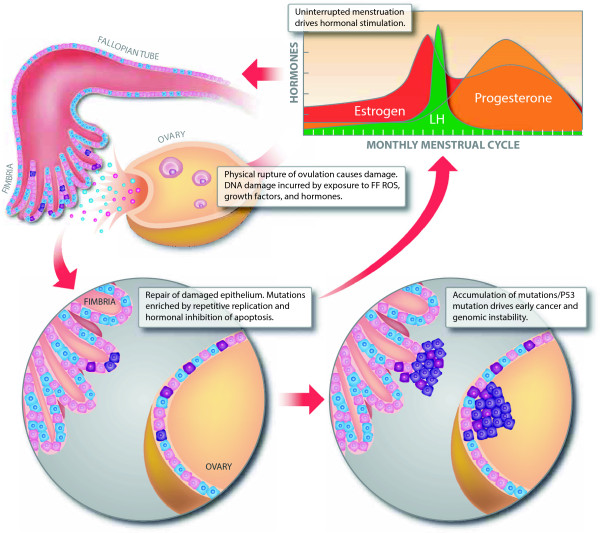
**Proposed mechanisms of HGSOC pathogenesis.** Incessant Ovulation Hypothesis: monthly physical damage from ovulation necessitates increased cell proliferation during repair, eventually leading to genomic instability. Gonadotropin hypothesis: Exposure to hormones released during ovulation inhibits natural apoptosis, uninterrupted hormonal fluctuations stimulate differentiation, proliferation, and ultimately malignant transformation.

Understanding the various cells of origin in ovarian cancer similarly informs our understanding of ovarian cancer pathogenesis. Several models have been posited to explain how epidemiological factors such as menstruation and ovulation may lead to ovarian cancer. A long standing hypothesis, often called the Incessant Ovulation Hypothesis, suggests that the repetitive wounding and healing of the ovarian surface epithelium and adjacent tubal epithelium that is induced by monthly ovulation increases cell proliferation and thus the likelihood of genomic instability which could lead to oncogenesis (Figure 1) [4]. Another hypothesis, known as the Gonadotropin hypothesis, implicates excessive direct and indirect stimulation of the ovarian surface epithelium by gonadotropins, leading to differentiation, proliferation, and ultimately malignant transformation (Figure 1) [5]. More recently, the Incessant Menstruation hypothesis suggests that repeated exposure to retrograde menstruation exposes the ovary and fallopian tube to reactive oxygen species and oxidative iron from the blood [8]. Lastly, several recent papers have focused on damage induced by inflammation-mediated factors found in the follicular fluid [9,10]. The recurring theme in all these hypotheses is the incessant ovulatory damage. This reinforces the importance of ovulation, but also makes it difficult to separate the impact of each hypothesis as they are physiologically interconnected. Women with altered steroid hormone levels, such as those with Polycystic Ovarian Syndrome (PCOS), tend to ovulate sporadically, while women who take oral contraceptives receive the protective benefits of both lowered gonadotropin levels and inhibited ovulation [5,11]. The average age of onset in ovarian cancer is postmenopausal, at age 63, when hormonal levels have shifted and ovulation has stopped [12]. Thus, an unanswered question that also remains is why menopause is so temporally important to the onset of the disease.

### Ovulation and follicular fluid

Mature human follicles reach approximately 23 mm in diameter, yielding upwards of 5 mL of follicular fluid (FF) [13]. During ovulation, FF is released and bathes the surrounding tissue, including the ovarian surface epithelium and fallopian tube (FT) fimbria proximal to the ovary. The debate over whether the ovarian surface epithelium (OSE) or the FT fimbria is the cell of origin for HGSOC is discussed in detail elsewhere [6,14]. For the purposes of this review, both tissues are exposed to the risk factors associated with ovulation. While there is robust epidemiologic data correlating total ovulatory events to ovarian cancer risk, a biological explanation remains elusive. One possibility recently proposed is that FF is genotoxic to exposed epithelia (OSE and FTE) and that repetitive exposure to this fluid can lead to mutations and alterations that drive neoplastic transformation [15]. The composition of this fluid, known to play a critical role in the development of the follicle, has profound reproductive and pathogenic implications.

FF is initially derived from, and is similar in composition to, thecal capillary serum [16-18]. As the follicle develops, granulosa cells produce large polysaccharides, hormones, and growth factors which cannot pass the 100 kDA follicle-blood barrier, causing an osmotic gradient which further increases FF volume [19,20]. Ultimately, mature follicles in unstimulated women can contain as much as 1,000 fold higher levels of estrogen and progesterone than the serum, whereas other hormones such as follicle stimulating hormone (FSH) are not differentially concentrated [21]. Other potentially harmful factors, such as reactive oxygen species (ROS), have a physiologic window; their presence is required for embryo formation but particularly high levels within the FF are associated with poor embryo formation in IVF patients [22]. While FF components include hormones, fatty acids, inflammatory factors, reactive oxygen species, and growth factors, it is unclear what role these potentially anti-apoptotic and mutagenic components play in ovarian cancer pathogenesis [9,10,23].

## Follicular fluid sources

Follicular fluid is often studied in the context of livestock reproduction, and abattoirs provide a readily available source of whole ovaries and fallopian tubes. While valuable research has been obtained using non-human models, the advantages of increased follicle volume and sample availability must be considered against the limited relevance of working with non-human FF [24]. Humans are the only known mammals to develop ovarian cancer naturally, and the levels of major hormones in cow and camel follicles is known to be several orders of magnitude lower than their human counterparts [25,26]. Domesticated laying hens, despite being oviparous, provide a unique model in that they are extremely susceptible to ovarian cancer and reflect several aspects of human disease [27]. Thus, a close examination of human FF, though harder to obtain and manipulate, is most relevant to studying the relationship between FF composition and ovarian cancer pathogenesis in humans.

Many recent studies have been conducted on the composition of human FF to assess conditions which are linked to ovarian cancer risk, as well as the efficacy and prognostic value of various *in vitro* fertilization (IVF) methods. This review gathers the most recent studies where human FF factors have been measured and comprehensively examines their fluctuation both under normal conditions and due to various conditions or treatments which are linked to HGSOC risk, and any role they may play in cancer pathogenesis (Table 1). Ultimately, developing a better understanding of FF and its hormonal composition will illuminate its potential role in ovarian cancer pathogenesis.

**Table 1:** FF factors and their role in follicle development and cancer pathogenesis

**Factor**	**Role in follicle development**	**Potential role in pathogenesis**	**Mouse knockout model effect**
*Estradiol*	Follicle development (specifically mid-follicular to pre-ovulatory phases) [28,29]	Direct proliferatory effect [32], free radical generation [32], epidemiological risk factor for breast and uterine cancers [30]	Failure to develop mature follicles [28]
*Progesterone*	End stage follicle development [28,29]	Progestin containing oral contraceptives decrease ovarian cancer risk [41]	Failure to ovulate [28]
*Androgens*	Stimulates early follicle development [28]	Unknown	Decrease in fertility, granulosa cell number [28]
*FSH*	Stimulation of primordial follicles, dominant follicle selection [45]	Hormonal regulator of estrogen, progestrone, testosterone, FSH and its signaling pathways highly expressed in OVCAR cell lines [46,51]	Failure to ovulate, failure of primordial follicles to mature [46]
*AMH*	Inhibits primordial follicle growth [56].	Promotes growth and differentiation [54], elevated in granulosa cell tumors [62]. Unlikely player in HGSOC as matched controls have no difference in serum AMH, no correlation to stage or prognosis [63]	Fertile but with shorter period of fertility [56]
*LH*	Supports thecal steroidogenesis, induces ovulation and corpus luteum formation [64]	Promotes angiogenesis in EOC through PI3K/AKT-mTOR pathway, inhibits apoptosis and cisplatin mediated apoptosis in EOC [69,70]	Atrophied ovaries, hypogonadism, malformed antral follicles, no corpus lutea [66]

## The role of hormones in follicle development, fertility, and cancer pathogenesis

### Estradiol

#### Function

One of the most prominent components of FF, estradiol (E2), is dominant between the mid-follicular and pre-ovulatory phases (Figure 1). Mouse models lacking E2 fail to develop mature follicles [28,29]. With regards to cancer, estradiol is also thought to have a mutagenic effect, particularly in female reproductive tissue where elevated blood levels of estrogens have been associated with an increased risk of breast and uterine cancer [30,31]. Since follicular fluid E2 concentrations can reach 1000 fold that of serum levels, estradiol may also play a role in HGSOC pathogenesis [21]. The two main oncogenic mechanisms proposed for estrogen are indirect stimulation of cell growth, leading to an increased risk of transcriptional errors, and direct generation of DNA damage, particularly through the production of free-radicals [32]. A detailed review of estrogen’s direct and indirect mutagenic effects can be found elsewhere [31].

#### Pathological and physiological conditions

Studying E2 levels in women with reproductive abnormalities gives insight into infertility as well as long term cancer risk. Surprisingly, women with PCOS, known to have elevated serum E2 levels, showed no difference in their FF E2 when compared to male factor infertility controls [33]. Similarly, no difference in FF E2 was observed in women with endometriosis and male factor infertility controls [34].

The role of infertility in HGSOC risk versus the role of confounding infertility treatments is currently poorly understood. Studies measuring hormone levels in women who required large amounts of stimulation to ovulate and control subjects undergoing unstimulated *In Vitro* Fertilization (IVF) found that while older age decreased FF E2 levels, young low responders were comparable to control donors [21]. Further studies measuring FF E2 levels in women undergoing standard IVF with low ovarian reserve confirmed that FF E2 levels decrease with age, but are higher in younger women with low ovarian reserve compared to control donors [35]. This is consistent with blood serum levels of estradiol, which decrease over a woman’s reproductive life cycle and are lowest at menopause [36]. In general, it appears that in IVF stimulation suppresses the level of FF E2 as measured in young, healthy egg donors [37]. Unlike its clear role in uterine physiology and pathology, estrogen’s contribution to ovarian cancer remains unclear and additional research is needed to define its role in this setting.

### Progesterone

#### Function

The most abundant hormonal component of FF, progesterone (P4) is critical for the end stages of follicle development and for ovulation [29]. Knockout mice lacking progesterone fail to ovulate [38]. The role of progesterone is difficult to discern as increased levels of estrogen induce the production of progesterone receptors, inextricably linking their functions and responses [39]. Compared to E2, cellular responses to progesterone are much more diverse and therefore harder to implicate [40]. The molecular role of progesterone in HGSOC pathogenesis is unclear, but cumulative intake of specific exogenous progestins has no impact on risk except when combined with estrogen in oral contraceptives [41].

#### Pathological and physiological conditions

In the general population, it appears that age rather than infertility impacts FF P4 levels, and that P4 levels rise as a woman ages. In a study comparing low responders to control donors, P4 levels were the same between younger donors and young low responders, but higher in older low responders [21]. Similarly, young women with a normal ovarian reserve had lower FF P4 levels than their low ovarian reserve counterparts or older women [35]. Where follicular estrogen remained constant in both endometriosis and PCOS patients, follicular progesterone levels were markedly decreased in both groups [33,34].

In young, healthy egg donor populations, it appears that stimulating egg production has no effect on the FF P4 when compared to unstimulated donors [37]. However, in patients undergoing IVF, the current evidence is conflicting. The role of progesterone, while generally considered protective in breast cancer, remains unclear in the context of ovarian cancer.

### Androgens

#### Function

Androgens, although predominantly involved in male development, are also expressed in the ovary and fallopian tube and play a critical role in early follicle development [42,43]. The role of androgens on follicle development is species dependent, and humans are the only mammal not known to exhibit a decrease in AR as the follicle matures [28,33,44]. Female mice lacking a functional androgen receptor (AR) are less fertile and have a shorter reproductive window [42,43].

#### Pathological and physiological conditions

Unlike estrogen and progesterone, testosterone levels in mature follicles seem resistant to fluctuation, as women with infertility or IVF treatments are remarkably similar to their control counterparts. Follicular testosterone levels, uniquely to humans, continue rise as the follicle develops [33,44]. In patients with unstimulated cycles, testosterone was not significantly affected by age or responder status, with all patients comparable to donor controls [21]. As expected, when the mature follicles of women with PCOS were compared to women with male factor infertility, follicular testosterone levels were significantly higher in women with PCOS [33]. Stimulation in egg donors only slightly elevates the levels of FF testosterone as compared to their unstimulated donor controls [37].

### Follicle stimulating hormone

#### Function

FSH stimulates the development of primordial follicles and, through a feedback loop, maintains and selects the dominant follicle [45]. Mutant mice lacking the FSH-receptor are anovulatory. This is likely due to the fact that FSH blocks apoptosis in preantral follicles [46,47]. FSH-R knockout mouse models also lack circulating estrogen and have significantly less progesterone, while serum testosterone levels are increased, suggesting that FSH is also involved in hormonal regulation [46].

FSH serum levels rise at puberty and peak at menopause [45-47]. In younger women, high levels of serum FSH are indicative of ovary feedback dysfunction and are symptomatic of low ovarian reserve [47,48]. A notable exception to this is PCOS, where serum FSH levels are abnormally low [49].

#### Pathological and physiological conditions

Although follicular FSH is infrequently measured, it appears to be generally resistant to change. While one study confirmed that both women approaching menopause and young women who responded poorly to stimulation had high levels of follicular FSH, another found that neither ovarian reserve nor age altered FF FSH levels [21,35]. Although levels of follicular FSH have yet to be measured in PCOS patients, they do not appear to be affected by endometriosis [34]. Surprisingly, it is one of the few follicular hormones unaltered by IVF stimulation [37].

New research has emerged showing FSH to be highly elevated in a number of solid tumors. FSH is thought to be beneficial for oncogenesis in that it increases angiogenesis via the Vascular Endothelial Growth Factor (VEGF) pathway [50]. Given the rise of serum FSH in menopausal women that corresponds to the typical onset of ovarian cancer, it is worth noting that FSH has been shown to inhibit apoptosis in a variety of ovarian cancer subtypes *in vitro*[51]. One of the signaling pathways regulated by FSH, OCT4, is also highly expressed in a variety of ovarian cancer cell lines and tumor samples, and has been shown to be involved in inhibiting apoptosis and inducing chemo-resistance, as well as stem cell regulation [52,53]. Less is known about the role of FSH within the follicle, but taken together these findings suggest that FSH may be a hormone of particular interest in the induction and growth of ovarian tumors.

### Anti-müllerian hormone

#### Function

Anti-Müllerian hormone (AMH), is a transforming growth factor produced by the granulosa cells and involved in both growth and differentiation [54,55]. In the ovary, it functions to inhibit primordial follicle growth. AMH null mice are fertile and have fully developed ovaries but a much smaller fertility window [56]. AMH serum levels increase significantly in early childhood, peak in the early 20s, then decline until becoming undetectable at menopause [55,57]. During the menstrual cycle, AMH decreases sensitivity to FSH, and is negatively correlated with both FSH and estradiol [58-61] It is generally agreed upon that AMH is a marker of follicle status and is not affected by fluctuations in other hormones [59].

#### Pathological and physiological conditions

Follicular levels of AMH closely mimic those of the serum, decreasing with age and decreased ovarian reserve, and negatively correlated with follicular FSH [35,61]. AMH has recently been identified as uniquely elevated in granulosa cell tumors, and is currently being evaluated as a marker to track the recurrence of this disease [62]. However, for the more common epithelial ovarian cancers, serum AMH does not appear to differ from that of age-matched controls, nor does it correlate with stage of tumor or prognosis [63].

### Luteinizing hormone

#### Function

Luteinizing hormone (LH) is produced by the anterior pituitary gland [64] and is responsible for supporting steroidogenesis within LH receptor positive thecal cells [65]. Mice who are deficient in LH are infertile, exhibit atrophied ovaries, and have low serum levels of estradiol and progesterone [66]. Surprisingly, serum FSH levels remain unaffected in these models [66]. Over the course of a woman’s lifetime, serum LH levels will rise, peaking at menopause [36,67,68].

#### Pathological and physiological conditions

While LH serum levels are known to rise at menopause [36,68], follicular fluid levels of LH remain surprisingly stable throughout the reproductive window. No difference was found in the LH follicular fluid levels between young and aging women in multiple studies [21,35]. Fertility status also appeared irrelevant to follicular LH levels, as low responders and women with reduced ovarian reserves of all ages had similar levels, even compared to their young, un-induced counterparts [21,35]. Another study comparing endometriosis patients to male infertility controls found no difference in follicular LH levels as well [34]. While comorbidities and aging do not seem to affect follicular LH levels, stimulation with rFSH and recombinant choriogonadotropin (rCG) significantly decreases LH levels within the follicle by approximately 100 fold [37].

Recently, LH has been implicated in epithelial ovarian cancer (EOC) as an enhancer of angiogenesis. Working through the PI3K/AKT-mTOR pathway, LH is capable of inducing VEGF, thereby implicating its involvement in cell growth, invasion, and migration [69]. LH has also been shown to upregulate survivin, inhibiting apoptosis in epithelial ovarian cancer [70]. Of particular interest is the fact that EOC cells treated with LH are much less sensitive to cisplatin induced apoptosis, suggesting a mechanism of drug resistance [70].

### Technical considerations

While the microenvironment is critical to early ovarian cancer pathogenesis, several technical problems have plagued efforts to study it in detail. Access to follicular fluid features prominently on this list, as do lack of proper controls. For women who have been induced in the course of infertility treatments, multiple mature follicles and manual egg retrieval mean follicular fluid supply is plentiful, but at the expense of altered hormone levels. Oophorectomies, which often accompany hysterectomies, would provide a valuable source of unadulterated follicles except that these are generally performed later in life when the ovaries have atrophied. Very few experiments, with a few valuable exceptions, recruit young female volunteers for egg retrieval, severely limiting our knowledge of baseline hormonal composition [20,36]. Thus one of the main goals in this field should be to determine the separate risk factors of nulliparity and conditions which lead to infertility.

## Conclusions

The hormonal profile of follicular fluid is complex and constantly in flux, changing dramatically over the course of follicle development, as well as throughout a woman’s lifetime depending on her age, health status and fertility. Understanding the hormonal microenvironment of ovulation is critical to establishing a molecular link between incessant ovulation and early ovarian cancer pathogenesis. While studying the role of hormones in ovulation is important, no less important is the role of hormones in the transition to menopause, when ovarian cancer is typically diagnosed. Why this disease so often manifests itself after risk factors such as ovulation have come to an end, and when supposedly protective progesterone levels have risen and likely damaging estrogen levels are low is a puzzle at the crux of the ovarian cancer problem [21,35]. In the efforts to understand disease onset, cessation of ovulation and the hormonal milieu accompanying it will be as important as ovulation itself.

Currently, one of the most promising leads providing a molecular basis for the contribution of FF to ovarian cancer pathogenisis was reported by Bahar-Shany et al. [15]. Using human fallopian tube *ex-vivo* cultures and pooled human FF, they found that exposing fallopian tube epithelium to FF stimulated inflammatory and DNA repair pathways and resulted in the upregulation of pro-angiogenic and pro-inflammatory interleukin 8. They noticed that follicular fluid exposure led to DNA double stranded breaks and, consequently, the stabilization of the tumor suppressor *TP53*. Early precursors of high grade serous ovarian cancer are also defined by their high expression of *TP53* and high levels of DNA damage, although in the vast majority of these cases *TP53* is also mutated, often with a gain of function mutation [71]. Understanding the link between the temporary induction of *TP53* in response to FF exposure and the aquisition of mutations in P53 in early precursor lesions will be key in the future of ovarian cancer research.

The role of hormones in the later stages of ovarian cancer is also a field ripe for study. The relationship between hormonal microenvironment and tumor is complicated, in no small part because the hormone receptors of many primary tumors and ovarian cancer cell lines have been inactivated either directly or indirectly. The lack of response to hormone signaling is evidenced by the largely unsuccessful use of hormonal therapy in ovarian cancer, especially compared to its sweeping successes in breast cancer [72,73].

In the fight against ovarian cancer, many factors beyond the hormonal milieu play a role, and follicular fluid is by no means the sole initiator of tumorigenesis. Other theories, including telomere shortening [74,75] have been proposed as well. Ultimately, providing the molecular link between epidemiologic risk factors and disease mechanisms will have broad implications not only for ovarian cancer, but for infertility and development as well.

## Abbreviations

AMH: Antimulerian hormone; AR: Androgen receptor; E2: Estradiol; EOC: Epithelial ovarian cancer; FF: Follicular fluid; FSH: Follicle stimulating hormone; FT: Fallopian tube; HGSOC: High grade serous ovarian cancer; IVF: *In vitro* fertilization; OSE: Ovarian surface epithelium; P4: Progesterone; PCOS: Polycystic ovarian syndrome; rLH: recombinant luteinizing hormone.

## Competing interests

The authors declare that they have no competing interests.

## Authors’ contributions

ME reviewed the relevant literature and wrote the body of the manuscript. RD provided significant guidance in drafting and critically revising the manuscript. Both authors read and approved the final manuscript.
